# Time to pathologic diagnosis of suspicious breast lesions: An institution‐based study in five Ethiopian hospitals

**DOI:** 10.1002/ijc.35436

**Published:** 2025-04-10

**Authors:** Friedemann Rabe, Sefonias Getachew, Clara Yolanda Stroetmann, Nikolaus Christian Simon Mezger, Tewodros Yalew Gebremariam, Bereket Berhane, Alex Mremi, Blandina Theophil Mmbaga, Pauline Boucheron, Valerie McCormack, Pablo Santos, Adamu Addissie, Eva Johanna Kantelhardt

**Affiliations:** ^1^ Global & Planetary Health Working Group Institute of Medical Epidemiology, Biostatistics and Informatics, Martin Luther University Halle‐Wittenberg Germany; ^2^ Department of Epidemiology and Biostatistics School of Public Health Addis Ababa University Ethiopia; ^3^ Department of Global Public Health Karolinska Institutet Stockholm Sweden; ^4^ Department of Pathology College of Health Sciences, Addis Ababa University Ethiopia; ^5^ Department of Pathology St Paul's Millennium Medical College Addis Ababa Ethiopia; ^6^ Pathology Department Kilimanjaro Christian Medical Centre Moshi Tanzania; ^7^ Kilimanjaro Clinical Research Institute Moshi Tanzania; ^8^ KCMC University Moshi Tanzania; ^9^ Environment and Lifestyle Epidemiology Branch International Agency for Research on Cancer Lyon France; ^10^ Department of Gynaecology Martin‐Luther‐University Halle‐Wittenberg Germany

**Keywords:** Africa, breast cancer, cancer diagnosis, early diagnosis

## Abstract

Most breast cancer (BC) patients in sub‐Saharan Africa are diagnosed at advanced stages. The World Health Organization's Global Breast Cancer Initiative Pillar II has a benchmark to diagnose BC within 2 months of the first contact with a health care provider (HCP). In this study, we interviewed 345 women who received a diagnostic workup of a suspicious breast lesion (eventually diagnosed as benign or malignant) at five Ethiopian hospitals in 2022. We assessed the length of the diagnostic journey encompassing the pre‐contact interval between the first experience of symptoms and the first HCP visit, and the post‐contact interval between HCP visit and diagnostic pathology procedures. We used negative binomial regression models to identify factors influencing these time intervals. The median pre‐contact interval was 2.8 months (interquartile range [IQR] 0.5–9.8). The median post‐contact interval was 1.7 months (IQR 0.6–3.9). Regarding the post‐contact interval, 55% of patients received their pathologic diagnosis within the recommended 2 months after the first HCP visit and met the Global Breast Cancer Initiative's benchmark. Increased travel times, limited social support, and consulting multiple HCPs before seeking pathology evaluation prolonged post‐contact intervals. Older patients (>45 years) and those referred for pathology evaluation during the initial HCP visit experienced shorter post‐contact intervals. Of all 345 women, 39% were diagnosed with BC. The relatively low proportion of women diagnosed within the recommended time frame makes it evident that increased awareness for BC, easily accessible diagnostic services, and specific training for HCPs are essential for the timely diagnosis of BC in Ethiopia.

## Graphical abstract

What's new?

The World Health Organization's Global Breast Cancer Initiative set the benchmark to diagnose breast cancer within 2 months of the first contact with a health care provider. This study analyzed the diagnostic journey of women with breast symptoms at five Ethiopian hospitals. Of the 345 women interviewed who received a diagnostic workup of a suspicious breast lesion, 55% received a pathologically confirmed diagnosis within the recommended 2 months, and 39% were diagnosed with breast cancer. Increased awareness among women and frontline healthcare workers, and efficient referral systems are essential to improving breast cancer care and survival in Ethiopia.

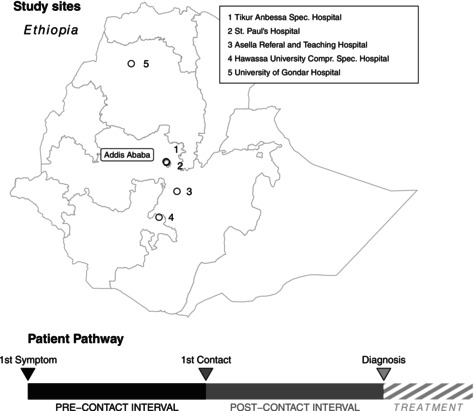

**Abbreviations**
BC: breast cancer
ETB: Ethiopian Birr
GBCI: Global Breast Cancer Initiative
HCP: health care provider
IQR: interquartile range
IRR: incidence rate ratios
KPI: key performance indicator
USD: United States Dollar
WHO: World Health Organization

## 1 INTRODUCTION

With more than 68,000 estimated breast cancer (BC) related deaths in 2022 and a rising incidence, BC has become a major burden on health systems in countries in sub‐Saharan Africa.^1^, ^2^ Advanced‐stage diagnosis of this potentially curable disease is common,^3^ with barriers to seeking medical advice and challenges during the diagnostic process identified as some of the key contributors to a delayed pathologic diagnosis. Time intervals of over 3 months between the onset of symptoms and the start of treatment are reportedly associated not only with an advanced stage at diagnosis but also with poorer treatment outcomes and worse survival probabilities in several countries,^4^ including Ethiopia.^5^, ^6^ In Ethiopia, the number of BC cases rises continually, and patient survival rates remain low.^6^, ^7^, ^8^ Long time intervals from symptom detection to the first HCP consultation concerning breast symptoms have been reported in different parts of the country.^9^, ^10^, ^11^ To our knowledge, the entire diagnostic journey of women with BC has solely been evaluated in Addis Ababa—Ethiopia's capital.^12^

The World Health Organization's Global Breast Cancer Initiative (GBCI) aims to reduce the population‐level age‐standardized BC‐related mortality rates by 2.5% per year globally. The initiative is structured around a three‐pillar framework: (i) health promotion to achieve a shift in TNM stage distribution towards earlier stages, (ii) timeliness of confirmatory diagnosis of breast lesions, and (iii) improving treatment completion. For the second pillar, a complete diagnostic workup of suspicious breast lesions within a 2‐month timeframe following the first presentation to a HCP has been defined as the key performance indicator (KPI).^13^

In this study, we analyzed the diagnostic journey of women with breast symptoms at five Ethiopian hospitals. We assessed the length of the diagnostic journey and its components, analyzed factors influencing time intervals, and determined the proportion of women who received timely breast diagnostics. Additionally, we evaluated the possibility of monitoring the above‐mentioned KPI set forth by the GBCI^13^ by interviewing patients on their pathway to care.

## 2 METHODS

### 2.1 Subjects and study design

This institution‐based study, consisting of a consecutive case series, was conducted between June 13th and November 30th, 2022, across the pathology departments of five tertiary‐level hospitals in Ethiopia. (Appendix S1 illustrates the locations of the five study hospitals.) Tikur Anbessa Specialized Hospital and St. Paul Millennium Medical College's Hospital are the two largest public hospitals, both located in the capital city, Addis Ababa. Tikur Anbessa serves as a national referral centre for oncology patients, with 19 of its 800 hospital beds dedicated to these patients. In total, 17 pathologists and six histotechnologists work at the hospital's pathology department. As of late, St. Pauls serves as an additional cancer care provider in Addis Ababa, with an inpatient capacity of 700 hospital beds and a pathology department that employs nine pathologists and nine histotechnologists. Hawassa University Comprehensive Specialized Hospital is a regional referral hospital, located 275 km to the south of Addis Ababa and provides surgical treatment as well as chemotherapy for cancer patients. The corresponding pathology department employs seven pathologists and eight histotechnologists. Assella Referral and Teaching Hospital is situated in the Oromia region, 175 km south of Addis Ababa and provides surgical care and chemotherapy for cancer patients. The hospital's pathology department employs four pathologists and five histotechnologists. Gondar University Hospital has provided oncology services since 2015. The hospital has 32 hospital beds dedicated to adult and paediatric oncology patients and employs eight pathologists and six histotechnologists. It serves a population of at least 30 million people in the Amhara region situated in the north of the country. The primary method for obtaining tissue samples from suspicious breast lesions is fine‐needle aspiration cytology supplemented, if necessary, by tissue collected through incisional biopsies or following complete tumour excision. Due to the high costs, core‐needle biopsies are performed only in select cases at Tikur Anbessa Specialized Hospital.

**FIGURE 1:**
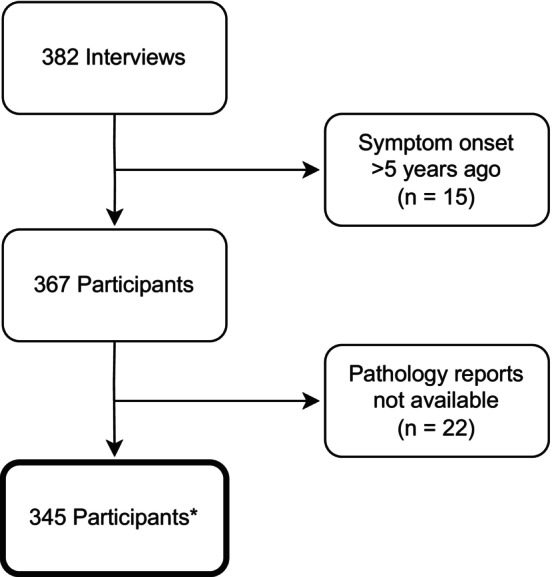
Flow chart of enrolment process and number of study participants. *Age > 18 years, female, no former diagnostic procedure for a similar condition.

Figure 1 illustrates the patient enrolment process, the total number of participants initially screened, the reasons for exclusions, and the final cohort included in the study. Over the course of our study, 444 women with breast symptoms suspicious of malignancy were consecutively referred from smaller hospitals, health centres, or private clinics for pathologic diagnosis and subsequent treatment initiation in the case of BC at one of the five study sites. All of them were approached at the pathology department and asked to participate. About 14% (*n* = 62) did not meet the inclusion criteria, which included female gender, age over 18 years, and no previous diagnosis or diagnostic procedures for a breast condition, and were therefore excluded. Of the 382 interviewed women, we excluded 16 participants because they remembered noticing breast symptoms for the first time >5 years previously and were therefore unlikely to have an incident condition. Pathology reports were available for 345 of these women.

The primary outcome of this study was the duration, in months, from symptom onset to a pathologically confirmed diagnosis of the breast lesion, irrespective of the result. Due to the varying reasons that may cause “delays” during the time before and after the first contact with an HCP, the two intervals, hereafter referred to as the *pre‐contact* and *post‐contact interval* (equivalent to GBCI KPI2), were measured separately. The *pre‐contact interval* spans the time from the first detection of symptoms to the first breast‐related HCP visit. The *post‐contact interval* includes the subsequent period leading up to the completion of the diagnostic workup.^14^

Data were collected from patients or their relatives using a questionnaire (Appendix S2) originally developed as part of the African Breast Cancer—Disparities in Outcome study.^15^ The questionnaire was adapted to the Ethiopian setting and translated into Amharic, the language mainly spoken in the regions where the study was conducted. To assess the time to diagnosis, participants were asked for the date of the first recognition of breast symptoms and the date they first presented to a professional HCP, that is, health centre, hospital, or private clinic. If participants could not recall exact dates, they were instead asked whether the event took place at the beginning, middle, or end of a certain month or year. Apart from the dates of these key events, the questionnaire captured experiences on the diagnostic pathway (e.g., first symptoms, outcome of the first HCP visit and type of health care facility visited), perceived obstacles to accessing diagnostic services, and a list of sociodemographic indicators (e.g., education, income, marital status, and health insurance). The questionnaire was piloted and adapted to the local context to ensure consistency and comprehensibility. Participants answered a face‐to‐face or telephone interview led by trained interviewers with a background in public health or nursing. Type (i.e. fine‐needle aspiration cytology, core‐needle biopsy, or incisional biopsy) and results of diagnostic procedures conducted were retrieved from pathology reports by the same interviewers. All data collected was entered into a REDCap database (www.project-redcap.org).

### 2.2 Statistics

All time intervals were calculated in months. We reported medians (with interquartile ranges, IQR) due to the non‐normal distributions of the interval lengths. A total of 58 explanatory variables were regrouped into seven broad categories, six according to the modified model of access by Penchansky and Thomas^16^, ^17^: availability, accessibility, adequacy, affordability, acceptability, and awareness, plus one additional category relating to the patient's medical history.

To reduce the number of variables to be modelled, we first assessed correlations among factors within each of the seven variable groups (Tables 2 and 3) using Spearman's correlation coefficients. Pairs of variables with a squared correlation coefficient above 0.6 were scrutinised, and one of the two variables was excluded from further analysis. This procedure was repeated until no strong evidence of a correlation remained within any variable group. The remaining variables were fitted to group‐specific unadjusted negative binomial regression models to identify factors associated with interval lengths. Variables that were significantly associated with the interval length (*p* ≤ .05) were included in the final models (Model 1: pre‐contact interval, Model 2: post‐contact interval). The final models were adjusted for sociodemographic characteristics such as age, education, marital status, and household income. In negative binomial regression, coefficients are typically exponentiated to obtain incidence rate ratios (IRRs). The IRR represents the multiplicative change in the expected count of events (response variable) that is associated with a one‐unit increase in the explanatory (predictor) variable.

As the results of the diagnostic workup (malignant or benign breast condition) were not known at recruitment, we performed a sensitivity analysis restricting the analysis to women diagnosed with BC only.

All analyses were conducted in R 4.1.0^18^ and Python (www.python.org).

## 3 RESULTS

### 3.1 Study participants

In all, 345 women were included in the analysis (Table 1). The median age at the time of the procedure for obtaining a sample for pathologic diagnosis was 34 years (IQR 26–42). Overall, 17% (*n* = 57) reported no formal education, and 40% (*n* = 137) were unemployed. Median monthly household income was 5000 ETB (97 USD), and 64% (*n* = 222) of the participants had no health insurance to cover the financial costs of the diagnostic process.

**TABLE 1:** Sociodemographic characteristics of all study participants (*n* = 345) and proportion of participants with pre‐contact interval <2.8 months (median of entire sample) and post‐contact interval <2 months (GBCI KPI 2).

	Total (*n* = 345)	Pre‐contact interval		Post‐contact interval	
		<2.8 months (median)	*p*‐value	<2 months (GBCI KPI 2)	*p*‐value
Study site			.131		.004
Tikur Anbessa	91 (26.4%)	49 (53.8%)		49 (53.8%)	
St. Pauls	88 (25.5%)	39 (44.3%)		55 (62.5%)	
Gondar	58 (16.8%)	21 (36.2%)		24 (41.4%)	
Hawassa	58 (16.8%)	31 (53.4%)		25 (43.1%)	
Assella	50 (14.5%)	20 (40.0%)		36 (72.0%)	
Age					
Median (IQR)	34 (26–42)				
Age groups			.999		.576
18–25	85 (24.6%)	40 (47.1%)		48 (56.5%)	
26–35	117 (33.9%)	56 (47.9%)		62 (53.0%)	
36–45	83 (24.1%)	37 (44.6%)		42 (50.6%)	
>45	60 (17.4%)	27 (45.0%)		37 (61.7%)	
Education			.003		.16
No formal education	57 (16.5%)	17 (29.8%)		26 (45.6%)	
Primary education^a^	102 (29.6%)	48 (47.1%)		60 (58.8%)	
Secondary education	116 (33.6%)	53 (45.7%)		59 (50.9%)	
Degree	70 (20.3%)	42 (60.0%)		44 (62.9%)	
Occupation			.066		.51
Formal employment	127 (36.8%)	70 (55.1%)		72 (56.7%)	
Housewife	99 (28.7%)	40 (40.4%)		48 (48.5%)	
No formal employment	38 (11.0%)	15 (39.5%)		20 (52.6%)	
Student	37 (10.7%)	15 (40.5%)		21 (56.8%)	
Other^b^	44 (12.8%)	20 (45.5%)		28 (63.6%)	
Marital status			.607		.368
Married	195 (56.5%)	91 (46.7%)		109 (55.9%)	
Never married	92 (26.7%)	40 (43.5%)		51 (55.4%)	
Divorced	30 (8.7%)	17 (56.7%)		18 (60.0%)	
Widowed	28 (8.1%)	12 (42.9%)		11 (39.3%)	
Monthly income					
Median (IQR)	ETB 5000 (3000–8000)				
	USD 97 (58–136)^c^				
Health insurance			.016		.007
Yes	123 (35.7%)	47 (38.2%)		55 (44.7%)	
No	222 (64.3%)	113 (50.9%)		134 (60.4%)	

Abbreviations: ETB, Ethiopian Birr; GBCI, Global Breast Cancer Initiative; IQR, interquartile range; KPI, key performance indicator; USD, United States Dollar.

^a^ Women who completed primary education (*n* = 68) and those who reported being able to read and write (*n* = 34) were combined into the ‘Primary Education’ category.

^b^ Women who reported to have their own business (*n* = 23) and retired women (*n* = 7) were combined into the ‘Other’ category.

^c^ Exchange rate of June 13th, 2022 (1 USD = 52 ETB, https://fxtop.com/).

### 3.2 Interval lengths

The median pre‐contact interval among 345 women was 2.8 (IQR 0.5–9.8) months (Table 2). Of them, 51% (*n* = 177) visited an HCP within 3 months, 13% (*n* = 44) between 3 and 6 months, and 36% (*n* = 124) more than 6 months after symptom discovery (Table 3).

**TABLE 2:** Median lengths (months) and interquartile ranges of pre‐contact interval, post‐contact interval, and ratio of the post‐contact interval to the entire diagnostic journey, according to study sites.

		Pre‐contact interval		Post‐contact interval		Proportion of post‐contact interval^a^	
Study site	*n* (%)	Median (months)	IQR (months)	Median (months)	IQR (months)	%	IQR (months)
All	345 (100%)	2.8	(0.5–9.8)	1.7	(0.6–3.9)	40	(12.7–79.1)
Tikur Anbessa	91 (26.4%)	2.2	(0.3–8.4)	1.8	(0.9–3.8)	41.6	(17.6–86.7)
St. Pauls	88 (25.5%)	2.3	(0.1–12.6)	0.8	(0.3–3.9)	35.4	(5.8–91.6)
Gondar	58 (16.8%)	6.0	(1.6–11.9)	2.2	(1.2–2.8)	38.8	(13.9–55.4)
Hawassa	58 (16.8%)	1.6	(0.6–6.9)	2.3	(1.1–8.2)	56.6	(30.1–89.1)
Assella	50 (14.5%)	3.4	(1.4–8.8)	0.7	(0.4–2.2)	22.2	(9.5–46.8)

Abbreviation: IQR, interquartile range.

^a^ Proportion of the post‐contact interval relative to the total diagnostic journey (i.e. the combined duration of pre‐contact and post‐contact intervals).

**TABLE 3:** Patients' characteristics and perceptions tested for association with length of pre‐contact interval.

Variable		Total proportion of all 345 (% in column‐%)	Proportion ≤3 months (% in row‐%)	Proportion >3 and ≤6 months (% in row‐%)	Proportion >6 months (% in row‐%)
All participants		345 (100%)	177 (51.3%)	44 (12.8%)	124 (35.9%)
Initial perception of symptoms					
Heard previously about BC^a^	Yes	225 (65.2%)	129 (72.9%)	31 (70.5%)	65 (52.4%)
	No	120 (34.8%)	48 (27.1%)	13 (29.5%)	59 (47.6%)
Sources of BC knowledge^a^					
TV or social media	Yes	171 (49.6%)	94 (53.1%)	25 (56.8%)	52 (41.9%)
	No	174 (50.4%)	83 (46.9%)	19 (43.2%)	72 (58.1%)
Relatives or friends	Yes	115 (33.3%)	71 (40.1%)	14 (31.8%)	30 (24.2%)
	No	230 (66.7%)	106 (59.9%)	30 (68.2%)	94 (75.8%)
Former hospital visit	Yes	34 (9.9%)	26 (14.7%)	4 (9.1%)	4 (3.2%)
	No	311 (90.1%)	151 (85.3%)	40 (90.9%)	120 (96.8%)
HCP	Yes	29 (8.4%)	17 (9.6%)	3 (6.8%)	9 (7.3%)
	No	316 (91.6%)	160 (90.4%)	41 (93.2%)	115 (92.7%)
Attributed first symptom(s) to cancer^a^ (NA = 1)	Yes	128 (37.1%)	77 (43.8%)	16 (36.4%)	35 (28.2%)
	No	216 (62.6%)	99 (56.2%)	28 (63.6%)	89 (71.8%)
Thought rapid HCP consultation necessary^a^ (NA = 2)	Yes	291 (84.3%)	157 (89.2%)	39 (88.6%)	95 (77.2%)
	No	52 (15.1%)	19 (10.8%)	5 (11.4%)	28 (22.8%)
Thought symptoms would resolve spontaneously^a^ (NA = 1)	Yes	204 (59.1%)	71 (40.1%)	35 (79.5%)	98 (79.7%)
	No	140 (40.6%)	106 (59.9%)	9 (20.5%)	25 (20.3%)
Thought BC is incurable^a^ (NA = 3)	Yes	183 (53.0%)	84 (47.7%)	27 (61.4%)	72 (59.0%)
	No	159 (46.1%)	92 (52.3%)	17 (38.6%)	50 (41.0%)
Thought over‐the‐counter medication sufficient treatment^a^ (NA = 3)	Yes	137 (39.7%)	51 (28.8%)	20 (46.5%)	66 (54.1%)
	No	205 (59.4%)	126 (71.2%)	23 (53.5%)	56 (45.9%)
Perceived severity of first symptom(s)^a^	Not serious	108 (31.3%)	36 (20.3%)	15 (34.1%)	57 (46.0%)
	Somehow serious	90 (26.1%)	42 (23.7%)	14 (31.8%)	34 (27.4%)
	Serious	78 (22.6%)	48 (27.1%)	11 (25%)	19 (15.3%)
	Very serious	69 (20.0%)	51 (28.8%)	4 (9.1%)	14 (11.3%)
Level of worry at symptom onset^a^ (NA = 1)	Not worried at all	106 (30.7%)	40 (22.6%)	15 (34.1%)	51 (41.5%)
	A little bit worried	76 (22.0%)	34 (19.2%)	7 (15.9%)	35 (28.5%)
	Worried	81 (23.5%)	46 (26.0%)	16 (36.4%)	19 (15.4%)
	Very worried	81 (23.5%)	57 (32.2%)	6 (13.6%)	18 (14.6%)
Knew HCP/hospital to visit for BC‐related problems^b^ (NA = 2)	Yes	129 (37.4%)	78 (44.1%)	13 (29.5%)	38 (31.1%)
	No	214 (62.0%)	99 (55.9%)	31 (70.5%)	84 (68.9%)
Lack of transportation to HCP/hospital^c^ (NA = 2)	Yes	84 (24.3%)	34 (19.2%)	10 (22.7%)	40 (33.1%)
	No	258 (74.8%)	143 (80.8%)	34 (77.3%)	81 (66.9%)
Concern about expenses^d^ (NA = 2)	Yes	121 (35.1%)	48 (27.1%)	20 (45.5%)	53 (43.4%)
	No	222 (64.3%)	129 (72.9%)	24 (54.5%)	69 (56.6%)
Difficulties making appointment^e^ (NA = 3)	Yes	152 (44.1%)	71 (40.1%)	19 (43.2%)	62 (51.2%)
	No	190 (55.1%)	106 (59.9%)	25 (56.8%)	59 (48.8%)
Conflicting responsibilities^e^ (NA = 3)	Yes	142 (41.2%)	47 (26.7%)	24 (54.5%)	71 (58.2%)
	No	200 (58.0%)	129 (73.3%)	20 (45.5%)	51 (41.8%)
Bad experiences during prior HCP visits^f^ (NA = 4)	Yes	52 (15.1%)	21 (11.9%)	6 (14.0%)	25 (20.7%)
	No	289 (83.8%)	156 (88.1%)	37 (86.0%)	96 (79.3%)
Fear of serious diagnosis^f^ (NA = 3)	Yes	202 (58.6%)	102 (57.6%)	28 (63.6%)	72 (59.5%)
	No	140 (40.6%)	75 (42.4%)	16 (36.4%)	49 (40.5%)
Fear of examination/procedure^f^ (NA = 3)	Yes	216 (62.6%)	111 (63.1%)	29 (65.9%)	76 (62.3%)
	No	126 (36.5%)	65 (36.9%)	15 (34.1%)	46 (37.7%)
Cultural/language barrier^f^ (NA = 3)	Yes	50 (14.5%)	20 (11.4%)	8 (18.2%)	22 (18.0%)
	No	292 (84.6%)	156 (88.6%)	36 (81.8%)	100 (82.0%)
Shame to talk about symptoms^f^ (NA = 2)	Yes	39 (11.3%)	17 (9.6%)	4 (9.1%)	18 (14.8%)
	No	304 (88.1%)	160 (90.4%)	40 (90.9%)	104 (85.2%)
Partner/family prohibited HCP visit^f^ (NA = 3)	Yes	7 (2.0%)	2 (1.1%)	2 (4.7%)	3 (2.5%)
	No	335 (97.1%)	175 (98.9%)	41 (95.3%)	119 (97.5%)
Patient history					
Prior routine breast examination (NA = 2)	Yes	29 (8.4%)	22 (12.5%)	2 (4.5%)	5 (4.1%)
	No	314 (91.0%)	154 (87.5%)	42 (95.5%)	118 (95.9%)
First symptom(s) noticed (several possible)					
Lump	Yes	287 (83.2%)	146 (82.5%)	40 (90.9%)	101 (81.5%)
	No	58 (16.8%)	31 (17.5%)	4 (9.1%)	23 (18.5%)
Pain	Yes	105 (30.4%)	52 (29.4%)	12 (27.3%)	41 (33.1%)
	No	240 (69.6%)	125 (70.6%)	32 (72.7%)	83 (66.9%)
Nipple retraction	Yes	36 (10.4%)	15 (8.5%)	4 (9.1%)	17 (13.7%)
	No	309 (89.6%)	162 (91.5%)	40 (90.9%)	107 (86.3%)
Discharge	Yes	31 (9.0%)	11 (6.2%)	5 (11.4%)	15 (12.1%)
	No	314 (91.0%)	166 (93.8%)	39 (88.6%)	109 (87.9%)
Ulceration	Yes	17 (4.9%)	3 (1.7%)	4 (9.1%)	10 (8.1%)
	No	328 (95.1%)	174 (98.3%)	40 (90.9%)	114 (91.9%)
Axillary mass	Yes	14 (4.1%)	9 (5.1%)	1 (2.3%)	4 (3.2%)
	No	331 (95.9%)	168 (94.9%)	43 (97.7%)	120 (96.8%)
Skin changes	Yes	7 (2.0%)	2 (1.1%)	3 (6.8%)	2 (1.6%)
	No	338 (98.0%)	175 (98.9%)	41 (93.2%)	122 (98.4%)

Abbreviations: BC, breast cancer; HCP, health care provider; NA, not answered.

^a^ Awareness.

^b^ Availability.

^c^ Accessibility.

^d^ Affordability.

^e^ Accommodation.

^f^ Acceptability.

The median length of the post‐contact interval (equivalent to GBCI KPI2) was 1.7 months (IQR 0.6–3.9), with 55% (*n* = 189) of the women undergoing a diagnostic procedure within 2 months (5%, *n* = 18 between 2 and 4 months, 22%, *n* = 76 after more than 4 months) of the first HCP visit (Tables 2 and 4). Overall, 135 women (39%) were diagnosed with BC (Table S3). Of them, 53% (*n* = 71) were diagnosed within 2 months of first presentation, with a median interval length of 1.8 months (IQR 0.7–4.5). Interval lengths of the women diagnosed with BC are displayed in Table S3.

**TABLE 4:** Patients' characteristics and perceptions tested for association with length of the post‐contact interval.

	Total: Proportion of all 345	Proportion ≤2 months	Proportion >2 and ≤4 months	Proportion >4 months
Variable	*n* (% in column‐%)	*n* (% in row‐%)	*n* (% in row‐%)	*n* (% in row‐%)
All participants	345 (100%)	189 (54.8%)	18 (5.2%)	76 (22.0%)
Setting and outcomes of first HCP visit				
Type of institution (NA = 1)				
Hospital	186 (53.9%)	105 (55.9%)	11 (61.1%)	43 (56.6%)
Health centre	97 (28.1%)	53 (28.2%)	4 (22.2%)	26 (34.2%)
Private clinic	60 (17.4%)	29 (15.4%)	3 (16.7%)	7 (9.2%)
Cancer centre	1 (0.3%)	1 (0.5%)	0 (0.0%)	0 (0.0%)
Private sector (vs. public)				
Private	71 (20.6%)	34 (18.0%)	4 (22.2%)	10 (13.2%)
Public	274 (79.4%)	155 (82.0%)	14 (77.8%)	66 (86.8%)
Recommendation/referral at first HCP visit				
Recommendation FNAC				
Yes	190 (55.1%)	121 (64.0%)	8 (44.4%)	34 (44.7%)
No	155 (44.9%)	68 (36.0%)	10 (55.6%)	42 (55.3%)
Recommendation breast imaging				
Yes	184 (53.3%)	99 (52.4%)	6 (33.3%)	32 (42.1%)
No	161 (46.7%)	90 (47.6%)	12 (66.7%)	44 (57.9%)
Referral to specialist				
Yes	146 (42.3%)	88 (46.6%)	9 (50.0%)	31 (40.8%)
No	199 (57.7%)	101 (53.4%)	9 (50.0%)	45 (59.2%)
Recommendation medication				
Yes	96 (27.8%)	41 (21.7%)	8 (44.4%)	27 (35.5%)
No	249 (72.2%)	148 (78.3%)	10 (55.6%)	49 (64.5%)
Recommendation to come back if symptoms aggravate				
Yes	27 (7.8%)	11 (5.8%)	1 (5.6%)	12 (15.8%)
No	318 (92.2%)	178 (94.2%)	17 (94.4%)	64 (84.2%)
Recommendation to see general practitioner				
Yes	14 (4.1%)	5 (2.6%)	0 (0.0%)	6 (7.9%)
No	331 (95.9%)	184 (97.4%)	18 (100%)	70 (92.1%)
Recommendation to do nothing				
Yes	9 (2.6%)	3 (1.6%)	1 (5.6%)	4 (5.3%)
No	336 (97.4%)	186 (98.4%)	17 (94.4%)	72 (94.7%)
Diagnosed with other condition than BC (NA = 4)				
Yes	90 (26.1%)	38 (20.4%)	4 (22.2%)	26 (34.2%)
No	251 (72.8%)	148 (79.6%)	14 (77.8%)	50 (65.8%)
Multiple HCPs visited				
Yes	94 (27.2%)	30 (15.9%)	10 (55.6%)	28 (36.8%)
No	251 (72.8%)	159 (84.1%)	8 (44.4%)	48 (63.2%)
Perceived barriers to access diagnostic procedure				
Perception of symptoms as not serious^a^ (NA = 2)				
Yes	158 (45.8%)	79 (42.2%)	7 (38.9%)	43 (56.6%)
No	185 (53.6%)	108 (57.8%)	11 (61.1%)	33 (43.4%)
Fear of serious diagnosis^a^ (NA = 5)				
Yes	253 (73.3%)	134 (72.4%)	14 (77.8%)	57 (75.0%)
No	87 (25.2%)	51 (27.6%)	4 (22.2%)	19 (25.0%)
Knew hospital offering diagnostic services^b^ (NA = 4)				
Yes	126 (36.5%)	69 (36.9%)	5 (27.8%)	28 (37.8%)
No	214 (62.0%)	118 (63.1%)	13 (72.2%)	46 (62.2%)
Area of residence^c^				
Urban	251 (72.8%)	145 (76.7%)	7 (38.9%)	49 (64.5%)
Rural	94 (27.2%)	44 (23.3%)	11 (61.1%)	27 (35.5%)
Lack of transportation to hospital^c^				
Yes	82 (23.8%)	33 (17.5%)	8 (44.4%)	28 (36.8%)
No	263 (76.2%)	156 (82.5%)	10 (55.6%)	48 (63.2%)
Long distance to hospital^c^ (NA = 8)				
Yes	80 (23.2%)	34 (18.4%)	9 (50.0%)	28 (38.4%)
No	257 (74.5%)	151 (81.6%)	9 (50.0%)	45 (61.6%)
Travel time to hospital (hours)^c^				
≤1 h	154 (44.6%)	99 (52.4%)	4 (22.2%)	28 (36.8%)
≤2 h	71 (20.6%)	37 (19.6%)	4 (22.2%)	12 (15.8%)
≤3 h	37 (10.7%)	13 (6.9%)	3 (16.7%)	12 (15.8%)
≤4 h	19 (5.5%)	11 (5.8%)	0 (0%)	5 (6.6%)
≤5 h	14 (4.1%)	7 (3.7%)	1 (5.6%)	5 (6.6%)
>5 h	50 (14.5%)	22 (11.6%)	6 (33.3%)	14 (18.4%)
Loss of income during hospital visit^d^				
Yes	94 (27.2%)	49 (25.9%)	8 (44.4%)	26 (34.2%)
No	251 (72.8%)	140 (74.1%)	10 (55.6%)	50 (65.8%)
Fear of costs of diagnostic procedure^d^ (NA = 8)				
Yes	225 (65.2%)	122 (65.6%)	13 (72.2%)	54 (74.0%)
No	112 (32.5%)	64 (34.4%)	5 (27.8%)	19 (26.0%)
Fear of financial hardship^d^ (NA = 6)				
Yes	112 (32.5%)	54 (28.9%)	10 (55.6%)	34 (46.6%)
No	227 (65.8%)	133 (71.1%)	8 (44.4%)	39 (53.4%)
No childcare available^e^				
Yes	76 (22.0%)	34 (18.0%)	4 (22.2%)	22 (28.9%)
No	269 (78.0%)	155 (82%)	14 (77.8%)	54 (71.1%)
Lack of lodging during hospital visit^e^				
Yes	56 (16.2%)	22 (11.6%)	5 (27.8%)	17 (22.4%)
No	289 (83.8%)	167 (88.4%)	13 (72.2%)	59 (77.6%)
Difficulties making appointment^e^ (NA = 6)				
Yes	126 (36.5%)	60 (32.1%)	9 (50.0%)	37 (50.7%)
No	213 (61.7%)	127 (67.9%)	9 (50.0%)	36 (49.3%)
Conflicting responsibilities^e^ (NA = 7)				
Yes	114 (33.0%)	53 (28.3%)	9 (50.0%)	36 (50.7%)
No	224 (64.9%)	134 (71.7%)	9 (50.0%)	35 (49.3%)
Employer did not allow time off^e^ (NA = 5)				
Yes	35 (10.1%)	19 (10.2%)	2 (11.1%)	5 (6.8%)
No	305 (88.4%)	168 (89.8%)	16 (88.9%)	68 (93.2%)
Work obligations^e^				
Yes	41 (11.9%)	23 (12.2%)	2 (11.1%)	8 (10.5%)
No	304 (88.1%)	166 (87.8%)	16 (88.9%)	68 (89.5%)
Lack of companion during hospital visit^f^				
Yes	24 (7.0%)	7 (3.7%)	1 (5.6%)	9 (11.8%)
No	321 (93.0%)	182 (96.3%)	17 (94.4%)	67 (88.2%)
Fear of procedure^f^ (NA = 3)				
Yes	261 (75.7%)	135 (72.2%)	14 (77.8%)	62 (81.6%)
No	81 (23.5%)	52 (27.8%)	4 (22.2%)	14 (18.4%)
Cultural/language barrier^f^ (NA = 6)				
Yes	35 (10.1%)	12 (6.4%)	4 (22.2%)	16 (21.9%)
No	304 (88.1%)	175 (93.6%)	14 (77.8%)	57 (78.1%)
Partner/family prohibited hospital visit^f^ (NA = 6)				
Yes	4 (1.2%)	1 (0.5%)	0 (0.0%)	2 (2.7%)
No	335 (97.1%)	185 (99.5%)	18 (100%)	71 (97.3%)

Abbreviations: BC, breast cancer; FNAC, fine needle aspiration cytology; HCP, health care provider; NA, not answered.

^a^ Awareness.

^b^ Availability.

^c^ Accessibility.

^d^ Affordability.

^e^ Accommodation.

^f^ Acceptability.

The length of the diagnostic journey differed between the study sites (Table 2). While the post‐contact interval was the shortest at Assella Referral and Teaching Hospital (median: 0.7 months, IQR: 0.4–2.2) and St. Paul Millennium Medical College's Hospital (median: 0.8 months, IQR: 0.3–3.9), patients at Hawassa University Comprehensive Specialized Hospital reported the longest post‐contact intervals (median: 2.3 months, IQR: 1.1–8.2). The post‐contact interval represented less than half of the diagnostic journey at four of the included hospitals, Hawassa University Hospital being the only exception. Differences in the distribution of the length of the post‐contact interval at the five study hospitals are shown in Figure S4.

### 3.3 Determinants of the pre‐contact interval length

While almost two thirds of the participants had prior knowledge about BC (65%, *n* = 225), about one third (37%, *n* = 128) initially considered BC as a possible cause of their symptoms. A routine breast examination, which included a palpatory clinical breast examination (6%, *n* = 21), sonography (2%, *n* = 9), and mammography (1%, *n* = 4) was reported by 8% (*n* = 29) before symptom onset. Nearly all participants (95%, *n* = 326) detected the symptoms themselves. The most common symptom reported was a “breast lump” (83%, *n* = 287), followed by “breast pain” (30%, *n* = 105).

**FIGURE 2:**
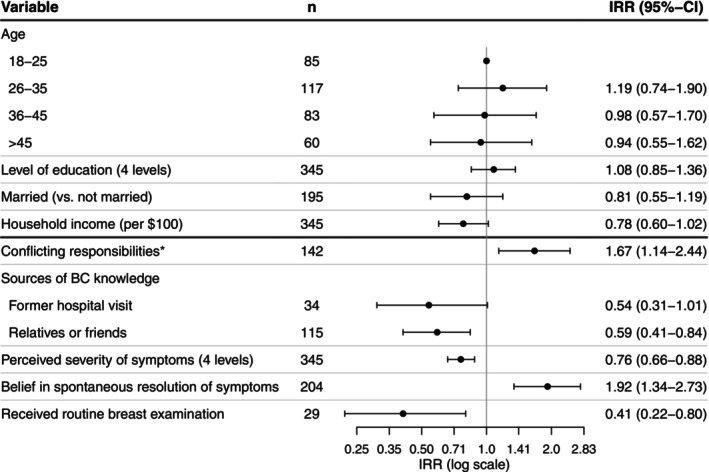
Model 1 presenting incidence rate ratios and 95% confidence intervals of factors associated with the length of the pre‐contact interval. BC, breast cancer; CI, confidence interval; IRR, incidence rate ratio. *Work, child care, care for a sick family member, studies.

Figure 2 presents the results of the negative binomial regression analysis modeling the effect of factors on the length of the pre‐contact interval. Women who had heard about BC from friends or relatives had shorter pre‐contact intervals (IRR: 0.59, CI: 0.41–0.84). Those who were informed during a former visit to a health care facility (i.e. posters, brochures at a hospital or health centre) tended to have shorter pre‐contact intervals (IRR: 0.54, CI: 0.31–1.01). Conflicting responsibilities, such as work or child care, were associated with longer pre‐contact intervals (IRR: 1.67, CI: 1.14–2.44). For each increase in the level of perceived seriousness of symptoms, the length of the pre‐contact interval decreased by 31% (IRR: 0.76, CI: 0.66–0.88), while it was almost twice as long for women who expected a spontaneous resolution of their symptoms (IRR: 1.92, CI: 1.34–2.73). Pre‐contact intervals were twice as short in women who reported previous routine breast examination (i.e. CBE or ultrasound or mammography) than in those who did not (IRR: 0.41, CI: 0.22–0.8).

### 3.4 Determinants of the post‐contact interval length

**FIGURE 3:**
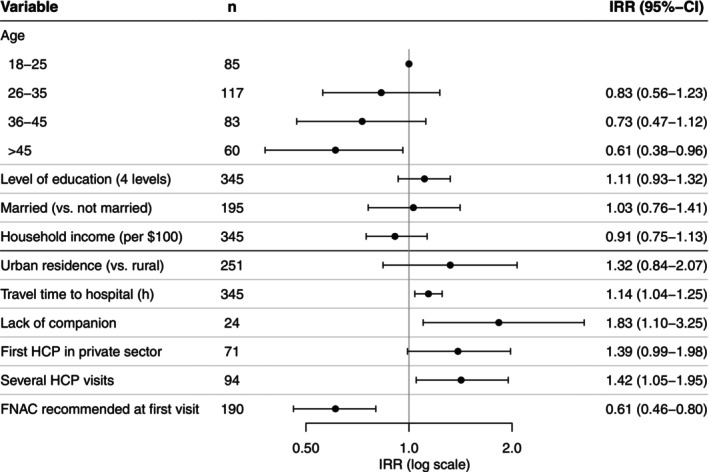
Model 2 presenting incidence rate ratios and 95% confidence intervals of factors associated with the length of the post‐contact interval. CI, confidence interval; FNAC, fine needle aspiration cytology; HCP, health care provider; IRR, incidence rate ratio.

Figure 3 presents the results of the negative binomial regression analysis modeling the effect of factors on the length of the post‐contact interval. The length of the post‐contact interval was associated with age, being shorter for older women (>45 years) than for women 18–25 years of age (IRR: 0.61, CI: 0.38–0.96). The interval length increased by 14% for each increasing hour that women had to travel to reach the hospital conducting the procedure (IRR: 1.14, CI: 1.04–1.25). Participants who lacked social support during the diagnostic process had longer post‐contact intervals (IRR: 1.83, CI: 1.01–3.25). The interval length decreased if participants were referred for further diagnostics (i.e. FNAC, biopsy) during the first HCP visit (IRR: 0.61, CI: 0.46–0.8) and increased when multiple HCPs were visited during the post‐contact interval (IRR: 1.42, CI: 1.05–1.95). There was no evidence of an association between urban (vs. rural) residence (IRR: 1.32, CI: 0.84–2.07) and the length of the post‐contact interval. However, the post‐contact interval tended to be longer in women who first visited an HCP in the private (IRR: 1.38, CI: 0.99–1.98) compared to the public sector.

## 4 DISCUSSION

In our cohort of 345 Ethiopian women with a suspicious breast lesion, the duration of the diagnostic journey from the first experience of symptoms to a pathologic diagnosis was often substantially longer than recommended by the WHO. A pathologically confirmed diagnosis within 2 months of the first visit to an HCP was received by only 55% (189) of women. The GBCI's goal to achieve post‐contact intervals shorter than 2 months for BC patients has not yet been achieved.^13^

### 4.1 Comparison with other studies

To our knowledge, there is only one study from the capital of Ethiopia assessing both pre‐ and post‐contact intervals. Gebremariam et al. reported interval lengths for both pre‐contact and post‐contact intervals. While the participants in our study reported pre‐contact intervals almost three times as long, the median post‐contact interval in our sample was about half as long.^12^ Other studies conducted in Ethiopia that focused on the pre‐contact interval and were conducted at regional hospitals in smaller cities reported slightly longer interval lengths than observed in our study.^9^, ^10^, ^11^ The variations in interval lengths are likely attributable to factors linked with the rural nature of the study area. These factors include fewer hospitals offering diagnostic services, less frequent public transportation, and limited access to education and information regarding BC.

Our results are similar to results from 1429 patients within the ABC‐DO study conducted in five African countries.^14^ While our pre‐contact interval was very similar to the one found in Zambia, the post‐contact interval was shorter and comparable to that in Namibia.^14^ Studies conducted in Cameroon,^19^ Rwanda,^20^ and Nigeria^21^ reported longer post‐contact intervals, while reports from South Africa^22^ and Mali^23^ reported shorter post‐contact intervals than the durations observed here.

A review that assembled data from 10 high‐income countries^24^ reported consistently shorter median post‐contact intervals compared to those observed in this study. Similarly, a study conducted across 12 European countries in 2011 found that median post‐contact intervals in two countries, Poland and Lithuania, were comparable to those reported here, while the remaining 10 countries exhibited longer intervals.^25^ By contrast, a study among BC patients in Malaysia reported a median post‐contact interval of 5.5 months,^26^ and a study in Peru recorded a mean post‐contact interval of 241 days.^27^

One possible explanation for the relatively short post‐contact intervals observed in Ethiopia is the widespread use of fine needle aspiration cytology as the primary method of pathological diagnosis. While fine needle aspiration cytology can be performed rapidly, it frequently does not yield definitive or reliable results.

### 4.2 Reasons for long intervals

Nearly two thirds of the women reported prior knowledge of BC before experiencing their first breast‐related symptoms, with 37% considering BC as a potential cause of their symptoms at that time. This contrasts with the findings of Gebremariam and colleagues, where only one third had prior knowledge and a mere 8% considered BC as a potential cause. Despite this increase, lacking BC awareness remains a fundamental factor contributing to long pre‐contact intervals among Ethiopian women. This applies not only to Ethiopia but also to other countries in sub‐Saharan Africa, where knowledge about BC is widespread, but many myths still exist.^28^

Being informed about BC at a former hospital visit or by relatives was associated with shorter pre‐contact intervals, while the belief that symptoms would resolve on their own was associated with longer pre‐contact intervals. Both factors related to prior BC knowledge and circumstances during symptom detection were identified as influential determinants of diagnostic journey duration.^29^ Notably, factors representing the five dimensions of healthcare access^16^ did not demonstrate a significant association with the length of the pre‐contact interval.

Long travel times to healthcare facilities and a lack of social support were drivers of longer post‐contact intervals in our study. A study in France, where social support was one of the main drivers of longer wait times before diagnosis and treatment initiation, reported similar findings.^30^ Barriers linked to accessibility and accommodation were found to impact post‐contact intervals, whereas factors related to availability, affordability, and acceptability showed no influence on the duration of the post‐contact interval. The former, especially accessibility, has been consistently identified in previous studies as a significant obstacle to a timely diagnostic workup.^29^ Women who were advised to see a specialist and seek pathologic confirmation of their breast lesion during their first HCP visit reported shorter post‐contact intervals. This supports the demand for strengthened referral pathways within the health system.^12^, ^14^, ^31^ The variations observed in the duration of the post‐contact interval at different study sites are likely caused by the diverse catchment areas, as well as the size and demographic characteristics of its catchment population. St. Paul Millenium Medical College's Hospital primarily serves the population of Addis Ababa, while Tikur Anbessa Specialized Hospital receives patients from a wider geographic area. By contrast, the three hospitals situated outside the capital city, while employing comparatively fewer specialized medical personnel such as pathologists and limited diagnostic capabilities, may be more easily accessible owing to their smaller catchment population and simpler referral processes.

Our study provides an initial estimate concerning one of the GBCI's KPIs in the second most populous country in sub‐Saharan Africa. By assessing the length of the diagnostic journey and its components, reported by many women with suspicious breast symptoms in Ethiopian hospitals, including tertiary care hospitals in the capital city Addis Ababa and smaller towns, it provides a comprehensive overview of the country's healthcare facilities. More than 85% of the women visiting the study sites for the diagnostic workup of their breast lesions over the course of the study could be enrolled, and direct interviews were conducted and analyzed instead of secondary data from patient files. One limitation of our study design is that participants were recruited at the pathology departments of the five included hospitals when their diagnostic journey was almost complete, which inadvertently excluded symptomatic women who did not progress that far. For those who did, this recruitment approach made it difficult to recollect exact dates and circumstances associated with the beginning of their diagnostic journey. On the other hand, this study proved to be a feasible approach to measure the post‐contact interval through short questionnaires at pathology centers to monitor the second KPI of the GBCI. However, our study does not provide information on the entire journey of BC patients. Geographical factors and detailed referral histories were not assessed. Additionally, the influence of the type of pathologic procedure conducted to diagnose BC and especially the timeliness of treatment initiation after diagnosis needs to be assessed in future studies.

## 5 CONCLUSION

Our study shows that the pathology service is an appropriate setting for assessing the timeliness of BC diagnosis. We found that the diagnostic journey for women with symptoms suggestive of BC in Ethiopia is shorter than that reported in many other countries in sub‐Saharan Africa. Nonetheless, it is noteworthy that only 55% of these women receive their diagnosis within the timeframe recommended by the GBCI. This relatively low proportion of women being diagnosed within the recommended period makes evident that increased awareness for BC among frontline healthcare workers, easily accessible diagnostic services for symptomatic women, and specific training for HCP are essential for timely diagnosis of BC in Ethiopia.

## AUTHOR CONTRIBUTIONS

**Friedemann Rabe:** Writing – original draft; writing – review and editing; visualization; conceptualization; methodology; data curation; investigation; formal analysis. **Sefonias Getachew:** Conceptualization; methodology; investigation; writing – review and editing; project administration. **Clara Yolanda Stroetmann:** Conceptualization; methodology; writing – review and editing. **Nikolaus Christian Simon Mezger:** Conceptualization; methodology; writing – review and editing. **Tewodros Yalew Gebremariam:** Conceptualization; project administration. **Bereket Berhane:** Project administration. **Alex Mremi:** Writing – review and editing. **Blandina Theophil Mmbaga:** Writing – review and editing. **Pauline Boucheron:** Writing – review and editing; conceptualization. **Valerie McCormack:** Conceptualization; writing – review and editing. **Pablo Santos:** Conceptualization; methodology; writing – original draft; writing – review and editing; formal analysis. **Adamu Addissie:** Conceptualization; project administration; resources. **Eva Johanna Kantelhardt:** Conceptualization; funding acquisition; writing – review and editing; resources; supervision; methodology.

## FUNDING INFORMATION

This work was supported by Else Kroener‐Fresenius Foundation Grant 2018_HA31SP (www.ekfs.de). This work was also supported by the German Ministry of Research and Education through the Network for Oncology Research in Africa (NORA), Grant 01KA2220B (https://www.gesundheitsforschung‐bmbf.de/en/rhissa‐research‐networks‐for‐health‐innovations‐in‐sub‐saharan‐africa‐8690.php), the Science for Africa Foundation to the Developing Excellence in Leadership, Training and Science in Africa (DELTAS Africa) program Grant No. Del‐22‐008 (https://scienceforafrica.foundation/deltas-africa) and the German Corporation for International Cooperation (GIZ) through the Developing International research collaboration in Ethiopia to support oncology at primary Health care levels (DINKNESH) program, Grant No. 81281915 (www.giz.de).

The funders played no part in the design of the study, data collection, analysis, decision‐making regarding publication, or manuscript preparation.

## DISCLAIMER

Where authors are identified as personnel of the International Agency for Research on Cancer/World Health Organization, the authors alone are responsible for the views expressed in this article and they do not necessarily represent the decisions, policy, or views of these organizations.

## CONFLICT OF INTEREST STATEMENT

All authors declared no potential conflict of interest.

## ETHICS STATEMENT

All women responding to the questionnaires provided their informed consent to participate in this study. The Ethics Committee of the Addis Ababa School of Public Health as well as the Research boards of the hospitals included have approved the study (Ref. No. SPH/1441/2022).

## Supporting information


**Appendix S1:** Supporting information.

## Data Availability

The data that support the findings of this study are available from the corresponding author upon request.
