# Supplementary material for: Time to pathologic diagnosis of suspicious breast lesions: An institution‐based study in five Ethiopian hospitals
Source: Int J Cancer. 2025 Apr 10;157(5):876–89. doi: 10.1002/ijc.35436 (PMC12232503; doi:10.1002/ijc.35436)

Supplementary material

Time to pathologic diagnosis of suspicious breast lesions: an institution-based study in five Ethiopian hospitals

Friedemann Rabe, Sefonias Getachew, Clara Yolanda Stroetmann, Nikolaus Christian Simon Mezger, Tewodros Yalew Gebremariam, Bereket Berhane, Alex Mremi, Blandina Theophil Mmbaga, Pauline Boucheron, Valerie McCormack, Pablo Santos, Adamu Addissie, Eva Johanna Kantelhardt

Table of contents

	Page
Supplementary Figure S1	2
Supplementary Material S2	3
Supplementary Table S3	9
Supplementary Figure S4	9

Supplementary Figure S1: Map of Ethiopia indicating the locations of the five included hospitals.

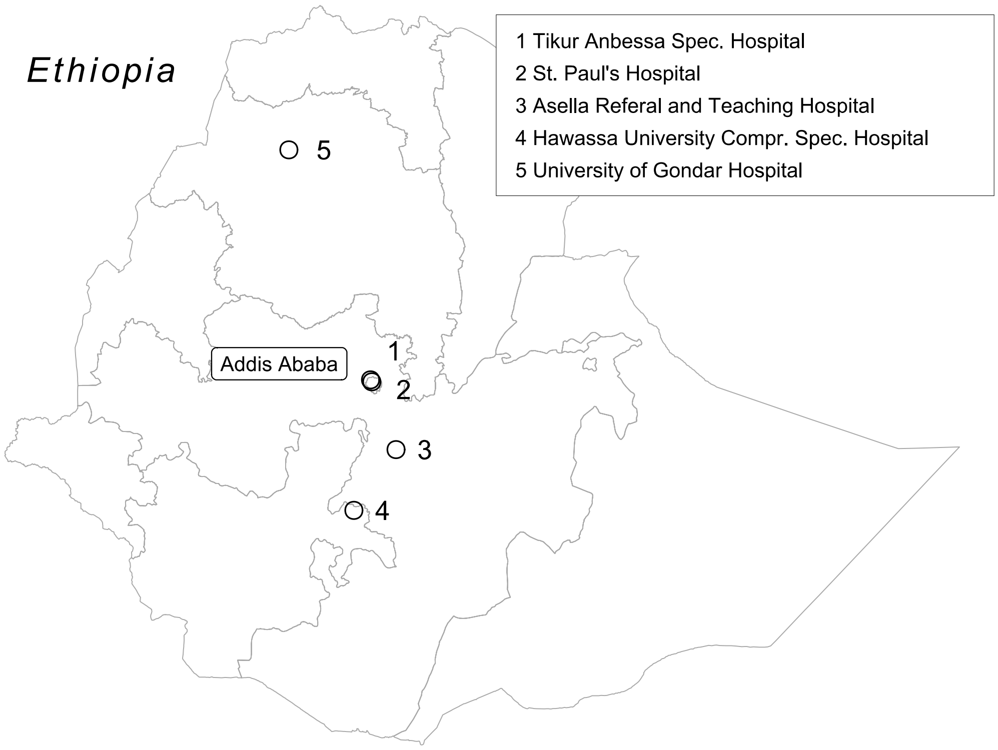

Supplementary Material S2: English version of the questionnaire used in the study. The original questionnaire was developed by McKenzie et al. as part of the African Breast Cancer – Disparities in Outcome study. It was subsequently adapted for the Ethiopian context and translated into Amharic.

DETERMINANTS OF TIME TO DIAGNOSIS OF SUSPICIOUS BREAST LESIONS

Inclusion criteria:		
Female	□ Yes	□ No
> 18 years old	□ Yes	□ No
Had or will have diagnostic procedure of suspicious breast lesion	□ Yes	□ No
First time to get diagnosed for suspicious breast lesion	□ Yes	□ No

If you ticked 'NO' in any of the four questions, stop the interview here.

StudyID	
1.01	Patients StudyID (4 digits):

SOCIO-DEMOGRAPHICS		
2.01	How old are you?	________ years
2.02	Which religion do you belong to?	O Ethiopian Orthodox<br>O Islam<br>O Protestantism<br>O Catholicism<br>O Other (specify): __________________________.
2.03	Do you live in a rural or urban area?	O Urban area<br>O Rural area
2.04	How much time did you need to get to the hospital where the procedure was/is done?	O < 30 minutes<br>O < 1 hour<br>O < 2 hours<br>O < 3 hours<br>O < 4 hours<br>O < 5 hours<br>O > 5 hours
2.05	What is your main occupation?	O Government employee<br>O Private employee<br>O Merchant<br>O Daily labourer<br>O Student<br>O Retired<br>O Housewife<br>O Unemployed<br>O Other (specify): __________________________.
2.06	What is your highest level of education?<br>*Please, tick the highest level of education that was actually completed.*	O Can't read or write<br>O Can read and write<br>O Elementary (Grade 1 – 8th)<br>O Secondary (Grade 9 – 12th)<br>O Degree and above
2.07	What is your current marital status?	O Married<br>O Single (never married)<br>O Separated/Divorced<br>O Widowed
2.08	What is your partner's educational level?<br>*Please, tick the highest level of education that was actually completed*	O Can't read or write<br>O Can read and write<br>O Elementary (Grade 1 – 8th)<br>O Secondary (Grade 9 – 12th)<br>O Degree and above

2.09	What is your partner's occupation?	O Government employee<br>O Private employee<br>O Merchant<br>O Daily labourer<br>O Student<br>O Retired<br>O Unemployed<br>O Other (specify): ___________________________.
2.10	How many children do you have?	____________ children
2.11	How many people live in your household?	____________ persons
2.12	Household income (per month)	____________ ETB
2.13	How do you pay for medical expenses?<br>*Multiple answers possible.*	O Health insurance<br>O Employing organization<br>O Out of pocket<br>O Family<br>O Free medical care<br>O Other (specify): ___________________________.
Only ask Q2.14 if the participant said that she has health insurance or health care is covered by employer.		
2.14	How much of the costs for the diagnostics of your breast symptoms are covered by the health insurance?	O Almost everything covered<br>O Only partially covered<br>O Not covered<br>O I don't know

AWARENESS			
Now all the questions I will ask you are about your knowledge and behaviour **before** noticing the first symptoms in your breast, so you have to imagine that you are sometime in the past, just before you discovered any changes.			
3.01	Have you ever heard of breast cancer before noticing the change in your breast for the first time?	O Yes	O No
3.02	Before you noticed the change in your breast did you know that the following symptoms could be signs for breast cancer? Please answer for each symptom separately:		
	- Breast lump	O Yes	O No
	- Pain/tenderness in the breast	O Yes	O No
	- Swelling in the breast	O Yes	O No
	- Bloody discharge from the nipple	O Yes	O No
3.03	Before you noticed the change in your breast have you received any of the following types of health education about breast cancer?		
	- programme/advertisement in the media (TV, radio, social media, or newspaper)	O Yes	O No
	- brochures or posters at a health centre/hospital	O Yes	O No
	- information from family, friends, colleagues	O Yes	O No
	- information by a doctor, nurse, public health worker	O Yes	O No
3.04	Did you ever have a breast exam before your symptoms?<br>*If 'no' go on to section 4*	O Yes	O No
3.05	Which tests did you receive?<br>*Multiple answers possible.*	O Palpation of the breast<br>O Mammography<br>O Ultrasound of the breast	
3.06	How often did you receive these tests?	Clinical breast exam: …… times<br>Mammography: …… times<br>Ultrasound: …… times	

SYMPTOM(S) RECOGNITION INTERVAL		
I will now ask you questions about the discovery of the symptoms in your breast.		
4.01	Who noticed the symptom first?	O You noticed it yourself<br>O Your husband (other relative) noticed first<br>O A health professional noticed first
4.02	Please try to remember the exact date you first noticed the symptom.<br>*If she does not remember the exact date use the algorithm to specify the date.*	d d m m y y y y
4.03	What was the first symptom you noticed?<br>*Multiple answers possible.*	O A lump (palpable)<br>O Nipple discharge<br>O Nipple retraction<br>O Skin changes (orange peel/dimpling)<br>O Ulceration<br>O Breast pain/tenderness<br>O Axillary mass/Mass under arm<br>O Other (specify): ________________________.
4.04	When you noticed this symptom, how serious did you think it was?	O Not serious at all<br>O Somehow serious<br>O Serious<br>O Very serious
4.05	To what extent were you worried then?	O Not worried at all<br>O A little bit worried<br>O Worried<br>O Very worried
4.06	Did you consider cancer as possible cause of the symptom?	O Yes O No

4.07	Please, answer the following questions with 'yes' or 'no'.<br>When you first noticed the symptom…		
a)	…did you know for such symptom(s)/findings, it is better to consult a doctor rapidly?	O Yes	O No
b)	…did you think the problem would disappear by itself?	O Yes	O No
c)	…did you think that cancer was an incurable disease?	O Yes	O No
d)	…did you think it was sufficient to treat the symptoms with ointments/over the counter medication?	O Yes	O No
e)	…did you wait to schedule your visit due to a bad experience with the medical services in the past?	O Yes	O No
f)	…were you afraid that the doctor would diagnose a serious disease?	O Yes	O No
g)	…were you worried about what the nurse/doctor might do (fear of pain, etc)?	O Yes	O No
h)	…did you think that the nurse/doctor would be unable to understand your language/culture?	O Yes	O No
i)	…did you feel embarrassed to talk about your symptoms to a nurse/doctor?	O Yes	O No
j)	…did you know which doctor/clinic you could visit to find help?	O Yes	O No
k)	…was it difficult to make an appointment at a clinic?	O Yes	O No
l)	…did you wait to schedule your visit because you had other priorities (children, work, sick family member, studies)?	O Yes	O No
m)	…did your husband/partner/family forbid you to go?	O Yes	O No
n)	…did you wait to schedule your visit because you were lacking transport to the doctor/clinic?	O Yes	O No
o)	…did you wait to schedule your visit because you were concerned that it would be too expensive?	O Yes	O No

ACCESS INTERVAL		
I will now ask you questions about your first visits to health facilities after noticing the symptom(s).		
5.01	When did you visit the health care professional for the first time?<br>*If she does not remember the exact date use the algorithm to specify the date.*	☐☐ ☐☐ ☐☐☐☐<br>d d m m y y y y
5.02	At which health facility did you see a doctor for the first time?<br>*Please, state the name of the institution if the answer is either 'hospital' or 'cancer centre'.*	O Health centre<br>O Hospital: ____________.<br>O Cancer centre: ____________.<br>O Private Clinic:
5.03	Was this doctor from the public or the private sector?	O Public<br>O Private
5.04	What did this doctor advise you to do concerning the breast symptoms?<br>*Multiple answers possible.*<br>*If the patient does not understand the term 'FNAC' please explain that it is the name of the procedure they have come for, where a sample of their breast lesion is taken to decide whether it is breast cancer. Please give this explanation if the patient does not understand the term on any other question during the interview.*	O To take a prescribed medication orally<br>O To see a general practitioner<br>O To see a specialist<br>O To have mammogram/ultrasound of the breast<br>O To have FNAC<br>O To apply local treatment (herbal medication/holy water)<br>O To come back if it got worse<br>O Other (specify):____________.<br>O Nothing
5.07	Which of the following did you do?<br>*Multiple answers possible.*	O I took a prescribed medication orally.<br>O I saw a general practitioner.<br>O I saw a specialist.<br>O I had mammogram/ultrasound of the breast.<br>O I applied a local treatment.<br>O I went back when it got worse.<br>O I went to see traditional healer.<br>O Nothing → *Pass on to section 6.*<br>O Other (specify): ____________.

DIAGNOSTIC INTERVAL		
I will now ask questions about the time between your first contact to a health care professional and today.		
6.01	Did you see any other doctors concerning your breast symptoms after that first visit, we just talked about before coming to this hospital?	O Yes O No
6.02	How many different doctors did you consult concerning your breast symptoms and how many health facilities did you visit to do so before coming here to receive FNAC?<br>*Doctors at this facility shall be included.*	_____ doctors at _______ facilities
6.03	So far, when you have been attending medical appointments for your breast cancer diagnosis, what major challenges did you face?<br>*Multiple answers possible.*	O No childcare available<br>O No transportation<br>O No lodging and/or food during hospital stay/clinic visits<br>O Loss of wages at the household<br>O Not getting time off work<br>O Not having a family member/friend accompany me<br>O COVID situation didn't allow movement<br>O Other (specify): ____________.

6.04	Please, answer the following questions with 'yes' or 'no'.<br>Before you came to the pathology for the first time to get your FNAC…		
	Did you get another diagnosis for the problem you have with your breast?	O Yes	O No
	Did you think that the symptom was not serious and therefore decided to wait before your diagnosis appointment?	O Yes	O No
	Were you afraid of what the doctors might tell you?	O Yes	O No
	Were you worried about what tests the nurse/doctor might do (fear of pain, anesthesia, surgery, etc)?	O Yes	O No
	Did you have difficulties in getting a rapid appointment (<2 weeks) at the hospital/clinic where to do diagnosis exams?	O Yes	O No
	Did you know which hospital to go to for the diagnostic procedure?	O Yes	O No
	Did you think the nurse/doctor would not understand your language or culture?	O Yes	O No
	Did you wait to schedule your visit because the doctor/clinic is far from your house or you didn't have transportation?	O Yes	O No
	Did you wait to schedule your visit because you had other priorities (children, work, sick family member, studies)?	O Yes	O No
	Did your husband/partner/family forbid you to go to the health care professional?	O Yes	O No
	Were you concerned that your employer will not give you time off to come here?	O Yes	O No
	Were you concerned the diagnosis procedure would be too expensive?	O Yes	O No
	Did you have financial problems to comply with the appointments and referral?	O Yes	O No

POSSIBILITIES FOR THE FUTURE		
We have almost finished our interview. I just want to add very few questions about possibilities to make access to breast diagnostics easier.		
7.01	What do you think would help women in the same situation like you on their way to diagnosis of their breast lesion? *Please specify:*<br>______________________________<br>______________________________	
7.02	Would you like to learn more about breast cancer?<br>*If the answer is 'No' pass on to Q7.04.*	O Yes O No
7.03	Where would you like to learn more about breast cancer?	O Media (TV, radio)<br>O Brochures, posters at health facilities<br>O Presentations at Churches<br>O Other (specify):<br>____________________.
7.04	Could you imagine to receive text messages on your mobile phone that inform you about breast cancer?	O Yes<br>O No<br>O Yes, but I do not have a mobile phone

The interview ends here.

Information from Pathology Report

Please repeat the participants StudyID
8.01 Patients StudyID (4 digits):

DATE of Diagnostic procedure:

d	d	m	m	y	y	y	y

Which diagnostic procedure was performed?

O FNAC (Fine needle aspiration cytology)
O CNB (Core needle biopsy)
O Incisional biopsy
O Tissue sample taken during surgery

DATE of Pathology Report:

d	d	m	m	y	y	y	y

Diagnosis as stated in the Pathology report:
Please note: "No result" if the diagnostic procedure was done without result. ____

Supplementary Table S3: Descriptive statistics of participants stratified by diagnosis as stated in the pathology report. The table presents median interval lengths, the proportion of the post-contact interval relative to the total diagnostic journey, and the proportion of patients who received a pathological diagnosis within 60 days of their first healthcare provider visit.

Diagnosis	All		Pre-contact interval		Post-contact interval		Prop. post-contact interval		< 60 days	
	n	%	median	IQR	median	IQR	Ratio (%)	IQR	%	n
All	345	100.0	2.8	(0.5:9.8)	1.7	(0.6:3.9)	40.0	(12.7:79.1)	54.8	189
inconclusive	17	4.9	1.2	(0.0:7.2)	1.1	(0.3:5.5)	30.0	(8.3:96.6)	58.8	10
malignant	135	39.1	4.0	(0.6:10.5)	1.8	(0.7:4.5)	38.1	(13.5:75.7)	52.6	71
benign	193	55.9	2.5	(0.6:10.1)	1.6	(0.5:3.6)	44.0	(13.0:75.3)	56.0	108

Supplementary Figure S4: Violin plots illustrating the distributions of interval lengths. The upper panel shows the distribution of pre-contact, post-contact, and total diagnostic journey intervals (i.e., the combined pre- and post-contact intervals). The lower panel depicts the distribution of post-contact interval lengths across the five study hospitals.

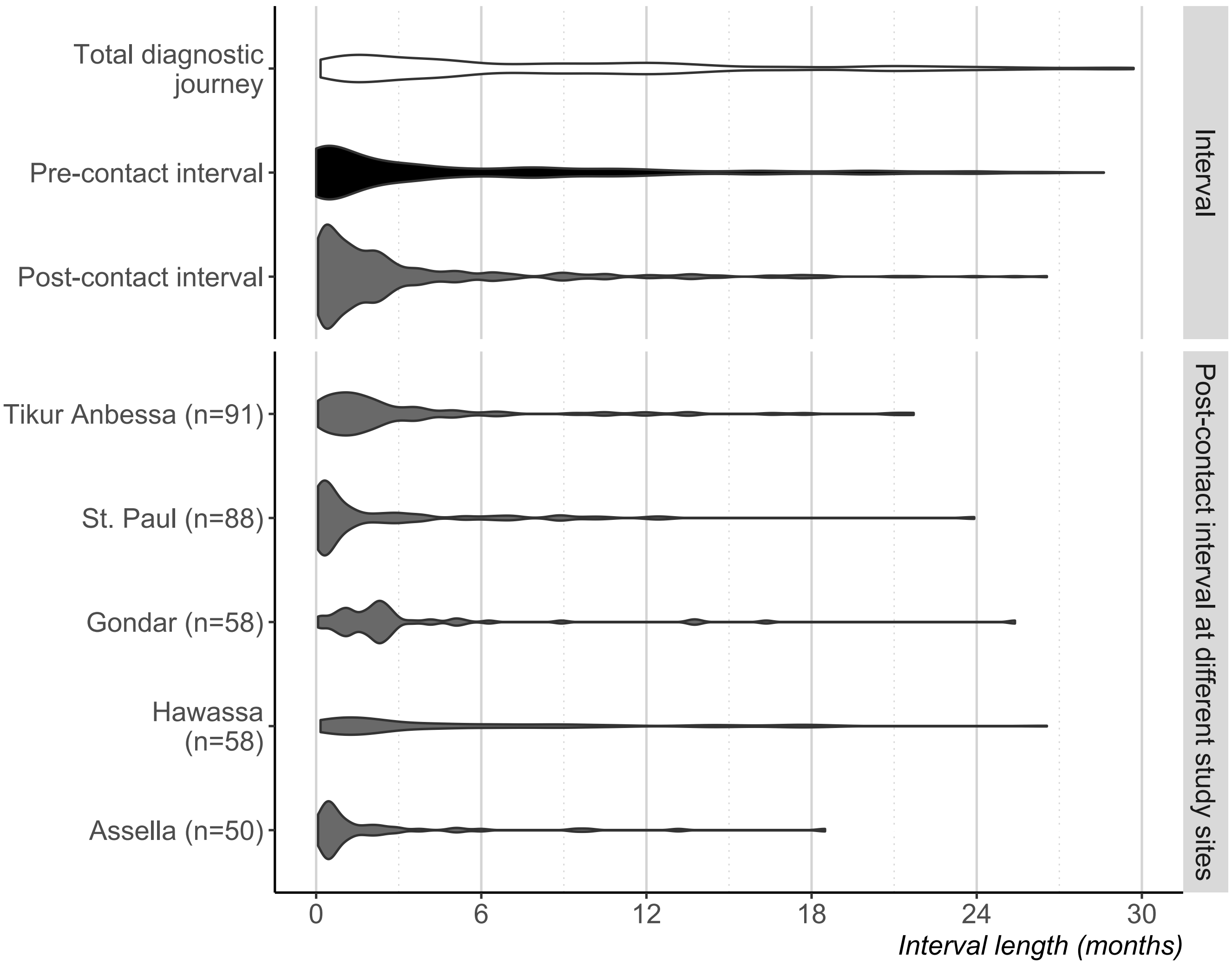

Supplement: Supplementary file 1 — Appendix S1: Supporting information. [file IJC-157-876-s001.pdf]
